# CCDC113 promotes colorectal cancer tumorigenesis and metastasis via TGF-β signaling pathway

**DOI:** 10.1038/s41419-024-07036-3

**Published:** 2024-09-11

**Authors:** Chenying Hou, Yanmei Yang, Peiwen Wang, Huimin Xie, Shuiling Jin, Liangbo Zhao, Guanghua Wu, Hao Xing, Hong Chen, Benyu Liu, Chunyan Du, Xiao Sun, Luyun He

**Affiliations:** 1 Tianjian Laboratory of Advanced Biomedical Sciences, Academy of Medical Sciences, Zhengzhou University, Zhengzhou, China; 2 Department of Oncology, the First Affiliated Hospital of Zhengzhou University, Zhengzhou, China; 3 School of Life Sciences, Zhengzhou University, Zhengzhou, China; 4 Laboratory Animal Center, Zhengzhou University, Zhengzhou, China; 5 Department of Pathophysiology, School of Basic Medical Sciences, Zhengzhou University, Zhengzhou, China

**Keywords:** Oncogenes, Metastasis, Tumour biomarkers

## Abstract

Colorectal cancer (CRC) is the second leading cause of cancer-related mortality worldwide. Although CRC patients’ survival is improved with surgical resection and immunotherapy, metastasis and recurrence remain major problems leading to poor prognosis. Therefore, exploring pathogenesis and identifying specific biomarkers are crucial for CRC early diagnosis and targeted therapy. CCDC113, a member of CCDC families, has been reported to play roles in ciliary assembly, ciliary activity, PSCI, asthma and early lung cancer diagnosis. However, the functions of CCDC113 in CRC still remain unclear. In this study, we find that CCDC113 is significantly highly expressed in CRC. High expression of CCDC113 is significantly correlated with CRC patients’ poor prognosis. CCDC113 is required for CRC tumorigenesis and metastasis. RNA-seq and TCGA database analysis indicate that CCDC113 is positively correlated with TGF-β signaling pathway. TGF-β signaling pathway inhibitor galunisertib could reverse the increased proliferation and migration ability of CRC cells caused by CCDC113 overexpression in vitro and in vivo. These results indicate that CCDC113 promotes CRC tumorigenesis and metastasis via TGF-β signaling pathway. In conclusion, it is the first time to explore the functions and mechanisms of CCDC113 in CRC tumorigenesis and metastasis. And CCDC113 may be a potential biomarker and therapeutic target for CRC intervention.

## Introduction

Colorectal cancer (CRC) is the second cause of cancer-related deaths and the third most common cancer worldwide [1]. Although surgical resection and immunotherapy have largely alleviated CRC patients’ clinical symptoms and prolonged their survival [2, 3], recurrence and metastasis remain the leading causes of death in CRC patients [4, 5]. Two-thirds of CRC patients develop distant metastasis and liver is the most common organ of CRC metastasis [6]. Gene mutation and accumulation of environmental risk factors are the main causes of CRC [7], and oncogenes play important roles in CRC tumorigenesis and metastasis. Five-year survival of CRC patients with advanced metastasis is only approximately 14% [8], so early diagnosis is the most effective way to improve CRC patients’ survival. Specific molecular biomarkers are important for early diagnosis and prognosis of CRC [9]. Therefore, exploring roles of key regulators in CRC tumorigenesis and finding specific CRC biomarkers are helpful to elucidate CRC pathogenesis, ultimately serving CRC clinical diagnosis and treatment.

CCDC (coiled-coil domain-containing) family contains approximately 180 genes from CCDC2 to CCDC201 [10], and they all have a highly conserved coiled-coil motif [11]. CCDC family members are widely involved in important biological functions including DNA recognition, protein scaffold, vesicles formation, ciliary motion and tumorigenesis and metastasis [12–14]. Coiled–coil domain containing 113 (CCDC113), also known as CFAP263, is located on human chromosome 16q21 and encodes a 377 amino acid protein. CCDC113 is identified to be a component of centrosome [15] and plays roles in ciliary beating regulation [16]. Bioinformatics analysis has shown that CCDC113 is associated with post-stroke cognitive impairment (PSCI) [17] and asthma [18, 19]. CCDC113 has also been predicted to be a biomarker for diagnostic detection of early lung cancer [20]. However, whether and how CCDC113 are involved in the regulation of CRC tumorigenesis and metastasis still remains largely unknown.

In this study, we identified CCDC113 is highly expressed in CRC through bioinformatics analysis in GEO CRC database. And high expression of CCDC113 is significantly correlated with CRC patients’ poor prognosis. CCDC113 knockdown significantly inhibits proliferation and migration of CRC cells, while CCDC113 overexpression has the opposite effects. In nude mouse subcutaneous xenograft tumor models and tail vein metastasis models, CCDC113 knockdown attenuates tumorigenesis and liver metastasis. While CCDC113 overexpression promotes tumorigenesis and liver metastasis. RNA-Seq and TCGA database analysis indicate that CCDC113 is positively correlated with TGF-β signaling pathway. Importantly, inhibition of TGF-β signaling pathway abolishes the oncogenic roles of CCDC113 overexpression in vitro and in vivo. Altogether, these findings reveal the role of CCDC113 in CRC tumorigenesis and metastasis, which may have important diagnostic and therapeutic implications in CRC.

## Results

### Screening of differentially expressed genes in CRC

**Fig. 1:**
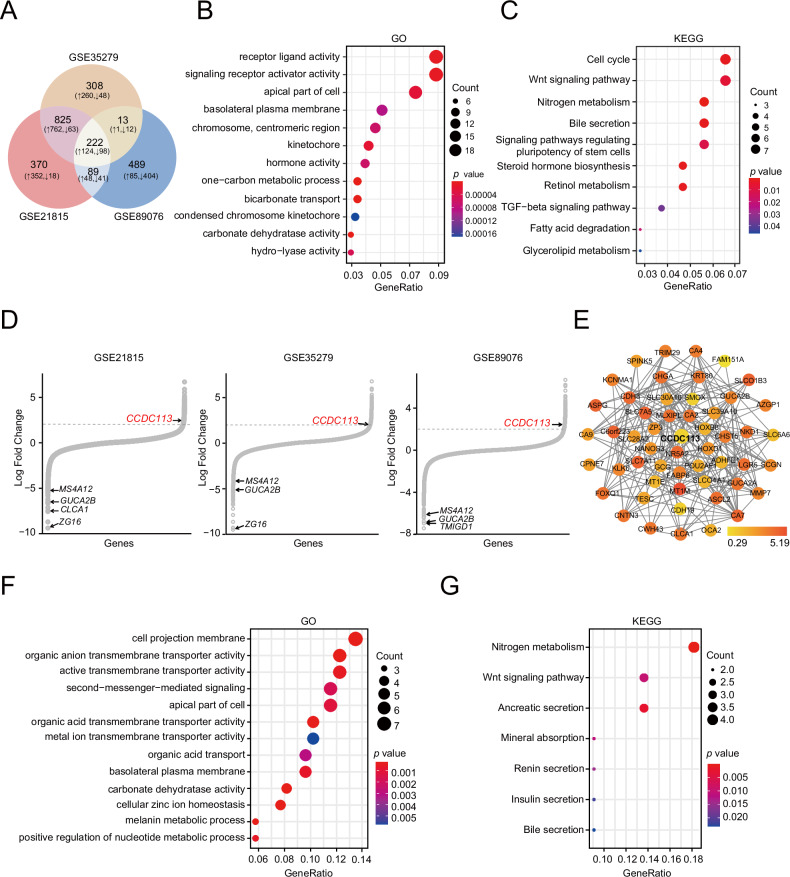
Screening of DEGs in CRC. **A** DEGs in GSE21815, GSE35279 and GSE89076 datasets were shown by Venn diagram. **B**, **C** GO (**B**) and KEGG (**C**) analysis of 222 common DEGs in (**A**). **D** DEGs in GSE21815, GSE35279 and GSE89076 datasets were shown by hockey-stick plots. **E** MCODE was used to extract 52 most highly interconnected clusters with CCDC113 from the string network of 222 common DEGs in (**A**). **F**, **G** GO (**F**) and KEGG (**G**) analysis of 52 CCDC113 related genes in (**E**).

To screen key regulators involved in tumorigenesis of CRC, three cohorts (GSE21815, GSE35279 and GSE89076) were selected for differentially expressed genes (DEGs) analysis between normal tissues and CRC tumor tissues. Finally, according to |log_2_FC | > 2 and adjusted *p* < 0.05, 222 common DEGs were identified, including 124 upregulated and 98 downregulated genes (Fig. 1A). Additionally, GO (Gene Ontology) and KEGG (Kyoto Encyclopedia of Genes and Genomes) enrichment analysis were conducted on 222 common DEGs, revealing predominant concentration on receptor ligand activity, signaling receptor activator activity (Fig. 1B) and Wnt signaling pathway and TGF-beta signaling pathway (Fig. 1C). DEGs in three cohorts (GSE21815, GSE35279 and GSE89076) were shown by hockey-stick plots (Fig. 1D). Through research literature and bioinformatics analysis, we specifically focused on CCDC113 among 124 common upregulated genes in three datasets, which has not been studied in CRC. We performed protein-protein interaction (PPI) network analysis by STRING online database (http://string-db.org) among 222 common DEGs and the sub-network showed 52 CCDC113 related genes clusters by MCODE plugin (Fig. 1E), implying CCDC113 as a central regulator. GO enrichment analysis of CCDC113 interacting proteins revealed significant enrichment in various transmembrane transporter activities, including organic anion transmembrane transporter activity and active transmembrane transporter activity (Fig. 1F). Also, these genes exhibited significant enrichment in both metabolism and Wnt signaling pathways (Fig. 1G). Conclusively, CCDC113 most likely plays a critical role in CRC tumorigenesis.

### CCDC113 is upregulated in CRC

**Fig. 2:**
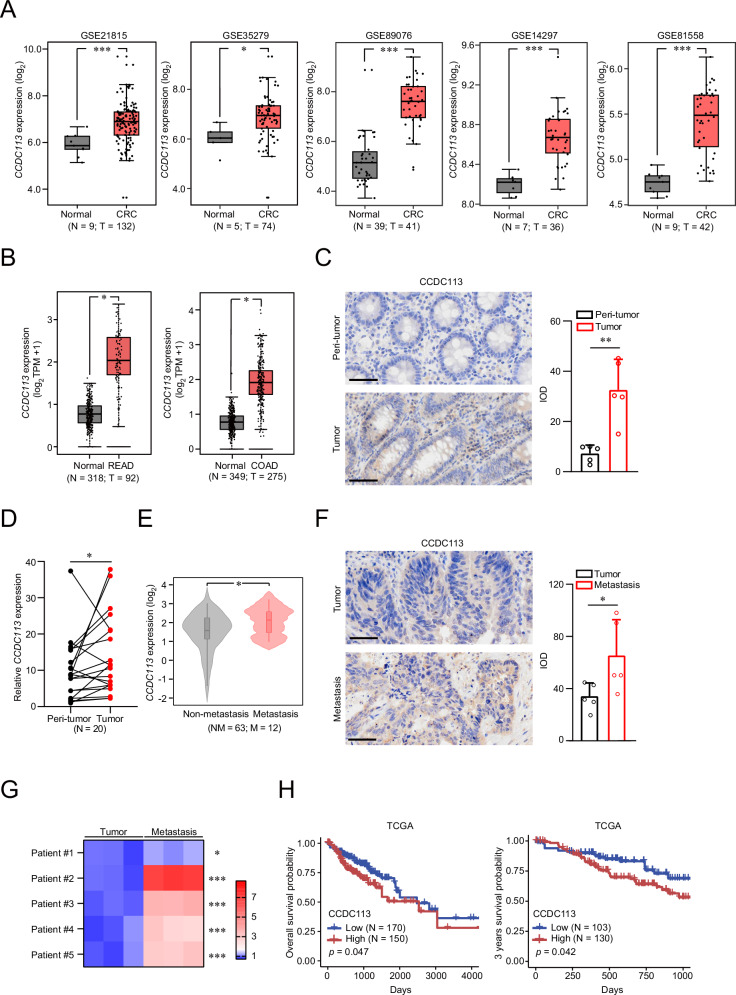
CCDC113 is upregulated in CRC. **A** CCDC113 expression in CRC tumor tissues according to GEO CRC datasets. **B** CCDC113 expression in COAD and READ according to TCGA and GTEx database. COAD: colon adenocarcinoma, READ: rectal adenocarcinoma. Boxplot lower extreme is first quartile, boxplot upper extreme is third quartile and median is shown with solid horizontal line in (**A**, **B**). **C** CCDC113 expression were detected by IHC staining in 5 pairs of CRC samples. Scale bars, 50 μm. The quantization (IOD values) of IHC staining was shown in right panel. IOD means integrated optical density. **D** CCDC113 expression in 20 pairs of CRC samples were detected by qRT-PCR. **E** CCDC113 expression in CRC non-metastasis tissues and CRC metastasis tissues according to TCGA database. **F** IHC staining of CCDC113 expression in 5 pairs of CRC tumor tissues and CRC liver metastasis tissues. Scale bars, 50 μm. **G** CCDC113 expression in 5 pairs of CRC samples were detected by qRT-PCR. **H** Kaplan-Meier analysis of CRC patients’ survival probability for overall survival and 3 years survival from TCGA databases. Data are presented as means ± SD. ***p < 0.001, **p < 0.01, *p < 0.05.

To verify the carcinogenic role of CCDC113, we reanalyzed its expression in GEO datasets (GSE21815, GSE35279, GSE89076, GSE14297 and GSE81558), TCGA and GTEx database. We found CCDC113 expression was higher in CRC tumor tissues than normal tissues (Fig. 2A). Moreover, CCDC113 expression was also higher in READ and COAD tissues (Fig. 2B). High expression of CCDC113 in CRC tumor tissues was verified by IHC staining (Fig. 2C) and qRT-PCR (Fig. 2D). Besides, CCDC113 upregulation was confirmed in CRC metastasis tissues compared to CRC non-metastasis tissues according to TCGA database (Fig. 2E). The higher expression of CCDC113 in CRC liver metastasis tissues than CRC tumor tissues was further confirmed by IHC staining (Fig. 2F) and qRT-PCR (Fig. 2G). Kaplan-Meier survival analysis revealed that high expression of CCDC113 was correlated with CRC patients’ poor survival (Fig. 2H). Conclusively, CCDC113 is highly expressed in CRC and positively correlated with CRC patients’ poor survival.

### CCDC113 knockdown inhibits CRC proliferation and migration in vitro

**Fig. 3:**
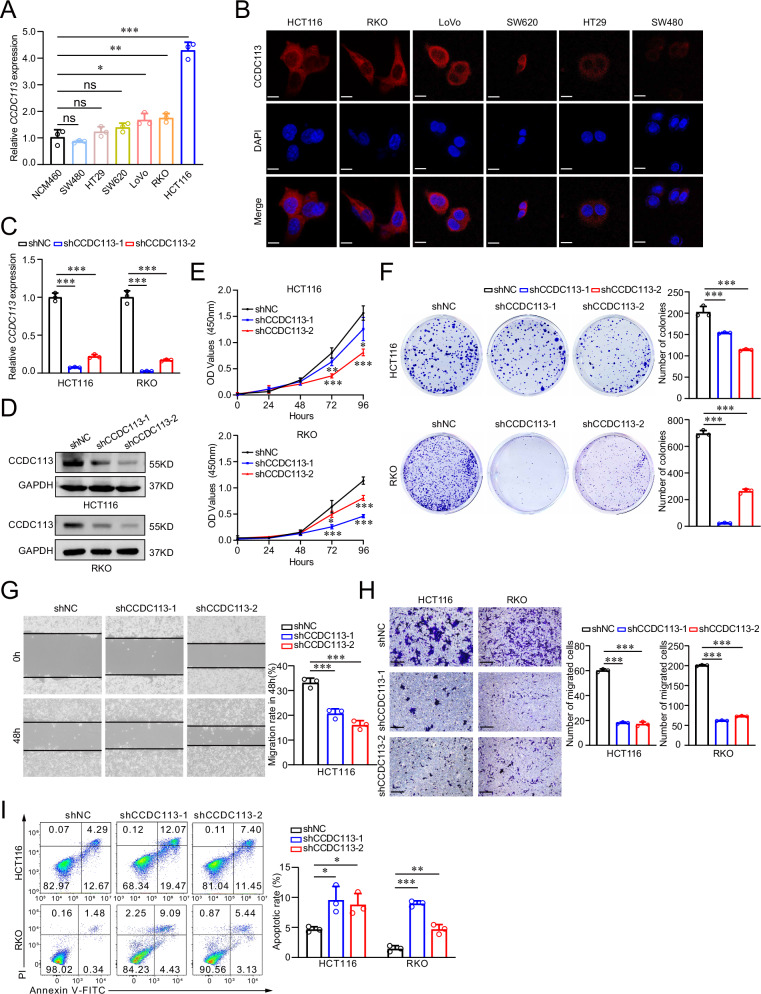
CCDC113 knockdown inhibits CRC cells proliferation and migration in vitro. **A** CCDC113 expression was detected by qRT-PCR in NCM460 (human normal colonic epithelial cell) and CRC cell lines. **B** Representative images of CCDC113 in CRC cells by immunofluorescence staining were shown. Scale bars, 10 μm. **C** CCDC113 expression were detected in CCDC113 knockdown HCT116 and RKO cells by qRT-PCR. **D** Western blotting detected CCDC113 expression in CCDC113 knockdown HCT116 and RKO cells. **E** CCK-8 assays detected cells viability of HCT116 and RKO cells after CCDC113 knockdown. **F** Colony formation assays detected proliferative abilities of HCT116 and RKO cells after CCDC113 knockdown. **G**, **H** Wound-healing assays (**G**) and transwell migration assays (**H**) detected migration abilities of HCT116 and RKO cells after CCDC113 knockdown. Scale bars, 100 μm. **I** FACS detected HCT116 and RKO cell apoptotic rate after CCDC113 knockdown. Representative images were shown in left panel and statistical results were shown in right panel. Data are presented as means ± SD.***p < 0.001, **p < 0.01, *p < 0.05, ns no significance.

To explore the role of CCDC113 in CRC, we detected CCDC113 expression in human normal colonic epithelial cell (NCM460) and CRC cells (SW480, HT29, SW620, LoVo, RKO, and HCT116). We found CCDC113 expression was higher in CRC cells than NCM460 cells (Fig. 3A). And CCDC113 expression was relatively higher in HCT116 and RKO cells among these CRC cells (Fig. 3A, B). So, we chose HCT116 and RKO cells for further study. Immunofluorescence staining showed that CCDC113 predominantly located in the cytoplasm of CRC cells (Fig. 3B). We designed two shRNAs and silenced CCDC113 in HCT116 and RKO cells (Fig. 3C, D). According to CCK-8 assays, CCDC113 knockdown significantly inhibited the viability of HCT116 and RKO cells (Fig. 3E). What’s more, colony formation assays revealed that proliferation abilities of HCT116 and RKO cells were also repressed in CCDC113 knockdown cells compared to control cells (Fig. 3F). Furthermore, wound-healing assays (Fig. 3G) and transwell migration assays (Fig. 3H) revealed that CCDC113 knockdown restrained the migration abilities of HCT116 and RKO cells. We also detected apoptotic cell rate after CCDC113 knockdown and found apoptotic cells rate was increased after CCDC113 knockdown (Fig. 3I). Conclusively, CCDC113 knockdown inhibits CRC cells proliferation and migration in vitro.

### CCDC113 knockdown inhibits CRC tumorigenesis and metastasis in vivo

**Fig. 4:**
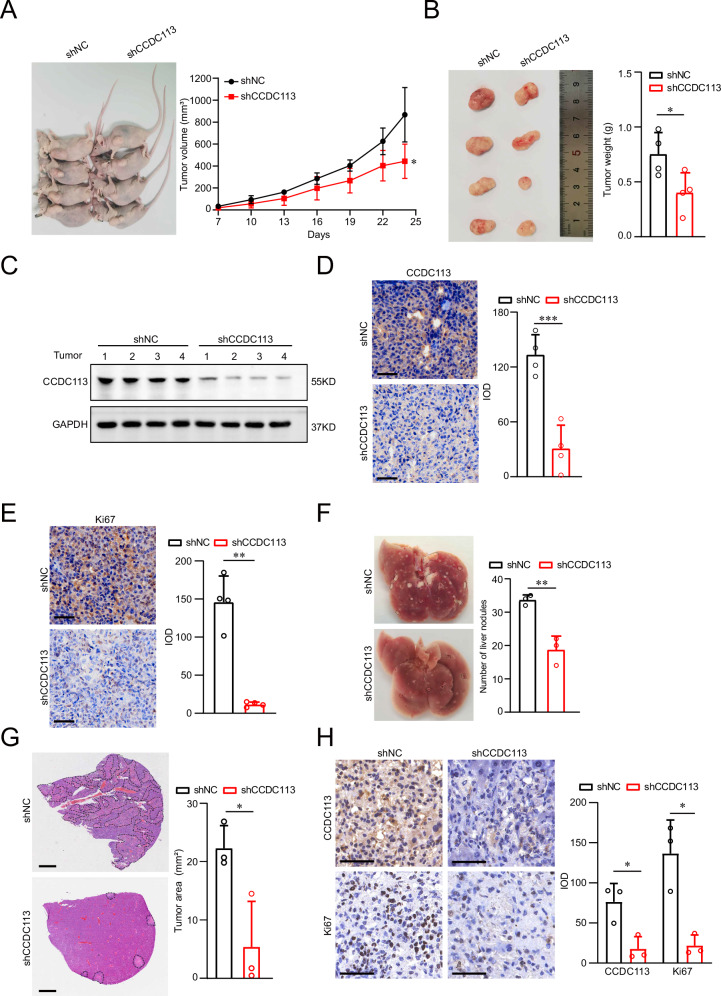
CCDC113 knockdown inhibits CRC tumorigenesis and metastasis in vivo. **A**, **B** CCDC113 knockdown HCT116 cells and control cells were subcutaneously injected into BALB/c nude mice for 24 days. Representative images of subcutaneous xenograft tumors were shown in left panel. Statistical results of tumor volumes (**A**) and tumor weights (**B**) were shown in right panel (*n* = 4 per group). **C** CCDC113 expression was detected by Western blotting in shNC and shCCDC113 of subcutaneous xenograft tumors. **D**, **E** IHC staining detected CCDC113 expression (**D**) and Ki67 expression (**E**) in subcutaneous xenograft tumors (*n* = 4 per group). Scale bars, 50 μm. **F** CCDC113 knockdown HCT116 cells and control cells were injected into tail vein of BALB/c nude mice for 42 days. Representative images of liver metastasis nodules were shown in left panel and statistical results were shown in right panel (*n* = 3 per group). **G** Liver metastasis nodules in (**F**) were analyzed by HE staining. Representative images were shown in left panel and statistical results were shown in right panel (*n* = 3 per group). Scale bars, 1 mm. **H** IHC staining detected CCDC113 and Ki67 expression in liver metastasis tissues (*n* = 3 per group). Scale bars, 50 μm. Data are presented as means ± SD. ***p < 0.001, **p < 0.01, *p < 0.05.

To explore the role of CCDC113 in CRC in vivo, we established subcutaneous xenograft tumor model. CCDC113 knockdown (shCCDC113) and control (shNC) HCT116 cells were subcutaneously injected into BALB/c nude mice. The tumor volumes and tumor weight in shCCDC113 group were significantly inhibited compared to shNC group (Fig. 4A, B), which was further confirmed by another independent shRNA (Fig. S1A, B). This reveals that CCDC113 knockdown attenuates xenograft tumor formation ability of CRC cells in vivo. Western blotting (Fig. 4C) and IHC staining (Fig. 4D) also showed that CCDC113 expression was significantly decreased in shCCDC113 group tumor tissues compared to shNC group in subcutaneous xenograft model. Moreover, Ki67 expression was sharply decreased after CCDC113 knockdown (Fig. 4E). To explore the metastasis ability of CCDC113 in vivo, we established tail vein metastasis model. shCCDC113 HCT116 cells and shNC HCT116 cells were injected into the tail vein of BALB/c nude mice for 42 days. We found liver metastasis nodules was significantly decreased in shCCDC113 group (Fig. 4F). HE staining also reached the same conclusion (Fig. 4G). Moreover, IHC staining showed that CCDC113 and Ki67 expression was significantly decreased in shCCDC113 liver metastatic nodules tissues compared to shNC group in tail vein metastasis model (Fig. 4H). Conclusively, CCDC113 knockdown inhibits CRC tumorigenesis and metastasis in vivo.

### CCDC113 overexpression promotes CRC proliferation and migration in vitro

**Fig. 5:**
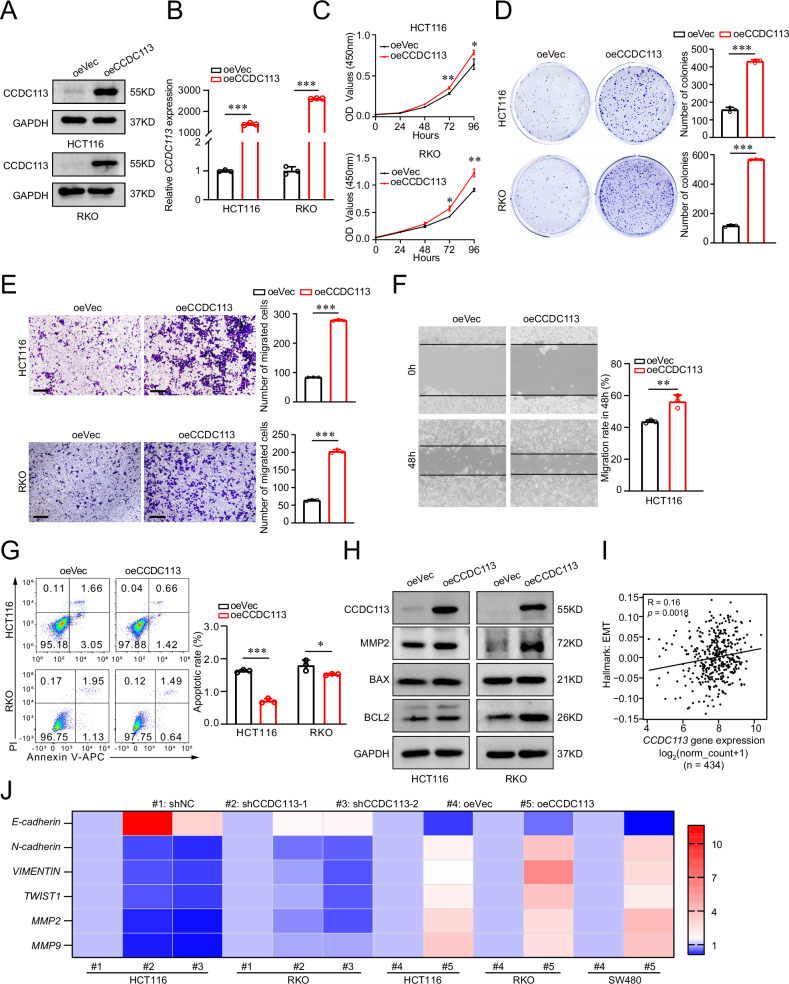
CCDC113 overexpression promotes CRC cells proliferation and migration in vitro. **A**, **B** Western blotting (**A**) and qRT-PCR (**B**) detected CCDC113 overexpression in HCT116 and RKO cells. **C** CCK-8 assays detected cell viability of HCT116 and RKO cells after CCDC113 overexpression. **D** Colony formation assays detected proliferation abilities of HCT116 and RKO cells after CCDC113 overexpression. Representative images were shown in left panel and statistical results were shown in right panel. **E** Transwell migration assays detected migration abilities of HCT116 and RKO cells after CCDC113 overexpression. Scale bars, 100 μm. Representative images were shown in left panel and statistical results were shown in right panel. **F** Wound-healing assays detected migration abilities of HCT116 after CCDC113 overexpression. Representative images were shown in left panel and statistical results were shown in right panel. **G** FACS detected HCT116 and RKO cell apoptotic rate after CCDC113 overexpression. Representative images were shown in left panel and statistical results were shown in right panel. **H** Western blotting detected expression of CCDC113, MMP2, BAX and BCL2 between CCDC113 overexpression HCT116 cells and control cells. **I** Correlation analysis between CCDC113 expression and EMT according to TCGA database. **J** Expression of EMT-related genes were detected by qRT-PCR. Data are presented as means ± SD. ***p < 0.001, **p < 0.01, *p < 0.05. Correlation *p* values were generated using the stat_cor function in the ggpubr package in R.

To further validate the role of CCDC113 in CRC, we constructed CCDC113 overexpression cell line (Fig. 5A, B). CCK-8 assays and colony formation assays indicated that CCDC113 overexpression significantly promoted proliferation abilities of HCT116 and RKO cells (Fig. 5C, D). Transwell migration assays and wound-healing assays showed that CCDC113 overexpression significantly promoted migration abilities of HCT116 and RKO cells (Fig. 5E, F). We also detected apoptotic cell rate after CCDC113 overexpression and found apoptotic cells rate was decreased after CCDC113 overexpression (Fig. 5G). Moreover, we detected the expression of CCDC113, MMP2 (associated with migration and invasion of cancer cells), BAX and BCL2 in CCDC113 overexpression cells and control cells. Western blotting showed the expression of MMP2 increased after CCDC113 overexpression (Fig. 5H), confirming its function on initiating metastasis. The expression of anti-apoptotic protein BCL2 increased significantly while the expression of proapoptotic protein BAX did not change distinctly after CCDC113 overexpression (Fig. 5H), which was consistent with above results. Correlation analysis showed that CCDC113 expression was positively correlated with EMT (epithelial-mesenchymal transition) (Fig. 5I). We also validated the positive correlation between CCDC113 expression and EMT related genes by qRT-PCR (Fig. 5J). Conclusively, CCDC113 overexpression promotes CRC proliferation and migration in vitro.

### CCDC113 overexpression promotes CRC tumorigenesis and metastasis in vivo

**Fig. 6:**
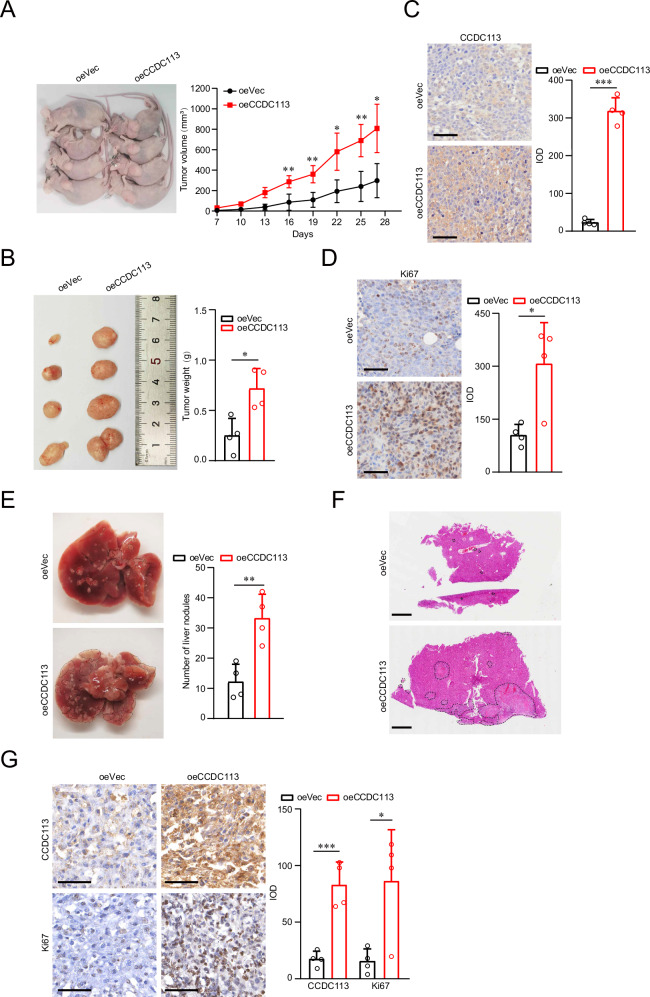
CCDC113 overexpression promotes CRC tumorigenesis and metastasis in vivo. **A**, **B** CCDC113 overexpression HCT116 cells and control cells were subcutaneously injected into BALB/c nude mice for 27 days. Representative images of subcutaneous xenograft tumors were shown in left panel. Statistical results of tumor volumes (**A**) and tumor weights (**B**) were shown in right panel (*n* = 4 per group). **C**, **D** IHC staining detected CCDC113 expression (**C**) and Ki67 expression (**D**) in oeVec group and oeCCDC113 group of subcutaneous xenograft tumors (*n* = 4 per group). Scale bars, 50 μm. **E** CCDC113 overexpression HCT116 cells and control cells were injected into tail vein of BALB/c nude mice for 28 days. Representative images of liver metastasis nodules were shown in left panel and statistical results were shown in right panel (*n* = 4 per group). **F** Liver metastasis nodules in (**E**) were analyzed by HE staining. Scale bars, 1 mm. **G** IHC staining detected CCDC113 and Ki67 expression in liver metastasis tissues (*n* = 4 per group). Scale bars, 50 μm. Data are presented as means ± SD. ***p < 0.001, **p < 0.01, *p < 0.05.

To confirm the role of CCDC113 in CRC in vivo, we established subcutaneous xenograft tumor model with CCDC113 overexpression HCT116 cells. CCDC113 overexpression (oeCCDC113) and control (oeVec) HCT116 cells were subcutaneously injected into BALB/c nude mice for 27 days. The tumor volumes (Fig. 6A) and tumor weight (Fig. 6B) of oeCCDC113 group significantly increased compared to oeVec group. IHC staining showed that the expression of CCDC113 (Fig. 6C) and Ki67 (Fig. 6D) obviously increased in oeCCDC113 group tumor tissues compared to oeVec group in subcutaneous xenograft tumor model. These results indicate that CCDC113 overexpression cells have a stronger xenograft tumor formation ability in vivo. To confirm CCDC113 metastasis ability in vivo, we established tail vein metastasis model of CCDC113 overexpression cells. oeCCDC113 HCT116 cells and oeVec HCT116 cells were injected into the tail vein of BALB/c nude mice for 28 days. We found liver metastasis nodules significantly increased in oeCCDC113 group (Fig. 6E). As confirmed by HE staining, oeCCDC113 group had more liver metastasis nodules than oeVec group (Fig. 6F). Moreover, IHC staining showed that CCDC113 and Ki67 expression was significantly increased in oeCCDC113 liver metastatic nodules tissues compared to oeVec group in tail vein metastasis model (Fig. 6G). These indicate that CCDC113 overexpression cells have a stronger liver metastasis ability. Conclusively, CCDC113 overexpression promotes CRC tumorigenesis and metastasis in vivo.

### CCDC113 promotes CRC tumorigenesis and metastasis via TGF-β signaling pathway

**Fig. 7:**
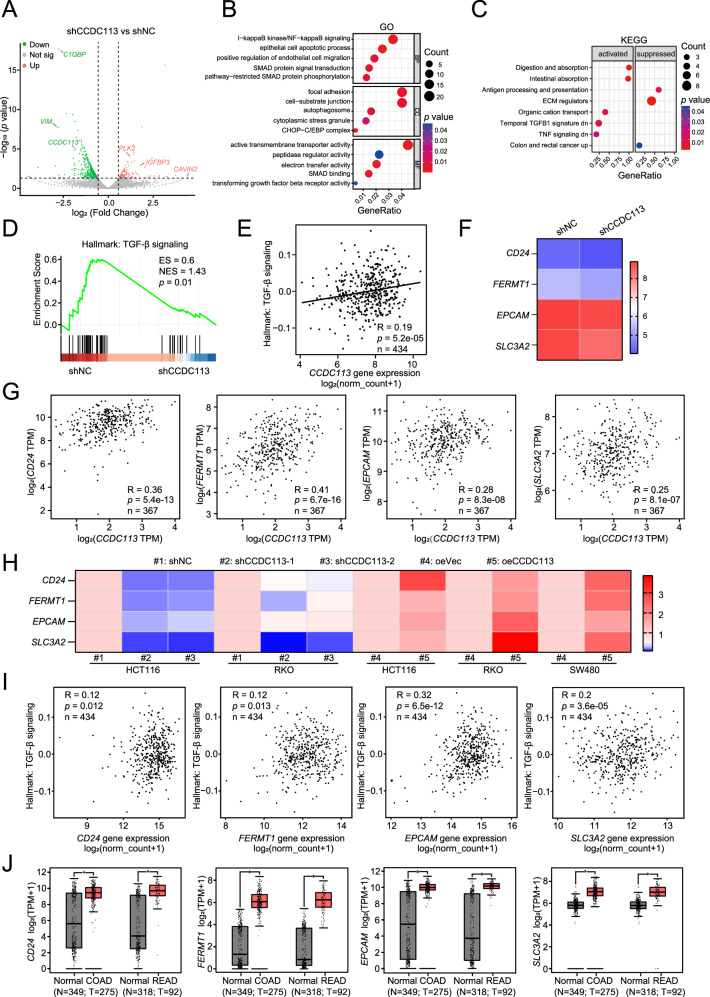
CCDC113 promotes CRC progression via TGF-β signaling pathway. **A** DEGs between shCCDC113 HCT116 cells and shNC cells were shown by volcano plot. **B** GO enrichment of DEGs in (**A**). BP Biological Process, CC Cell Component, MF Molecular Function. **C** KEGG pathway enrichment analysis of DEGs in (**A**). **D** GSEA of RNA-seq data between shCCDC113 HCT116 cells and shNC cells. **E** Correlation analysis between CCDC113 expression and TGF-β signaling pathway according to TCGA database. **F** Expression of CD24, FERMT1, EPCAM and SLC3A2 in shCCDC113 HCT116 cells and shNC cells according to RNA-seq data. **G** Correlation analysis of CCDC113 expression with CD24, FERMT1, EPCAM and SLC3A2 expression in CRC according to TCGA database. **H** Expression of CD24, FERMT1, EPCAM and SLC3A2 in CCDC113 knockdown and overexpression CRC cells were detected by qRT-PCR. **I** Correlation analysis of CD24, FERMT1, EPCAM and SLC3A2 expression with TGF-β signaling pathway according to TCGA database. **J** Expression of CD24, FERMT1, EPCAM, and SLC3A2 in CRC tissues according to TCGA and GTEx database. Correlation *p* values were generated using the stat_cor function in the ggpubr package in R.

**Fig. 8:**
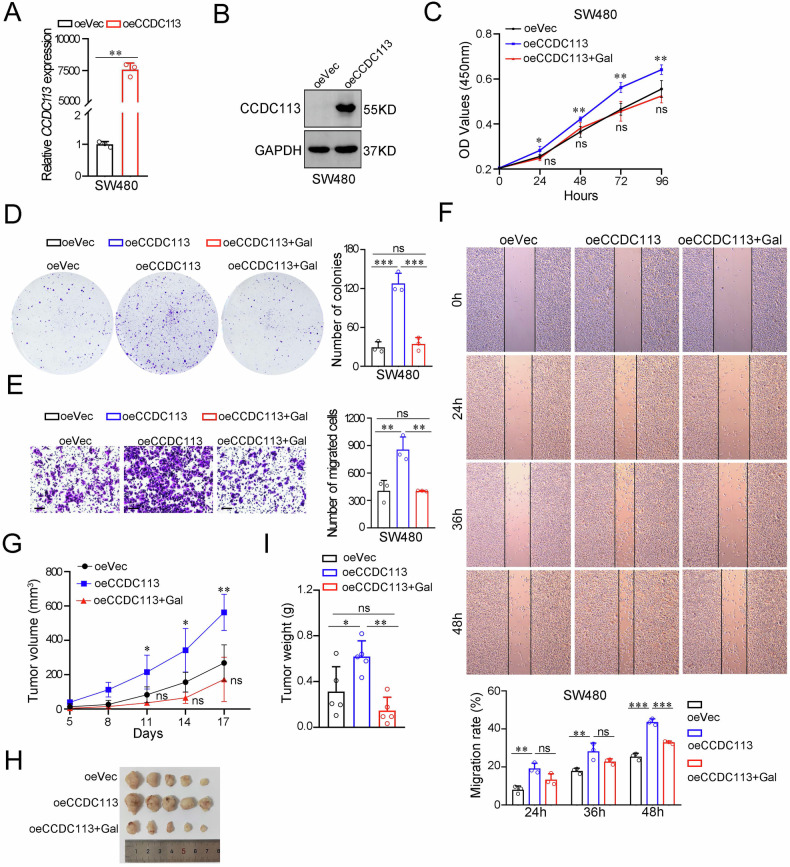
Galunisertib reverses increased proliferation and migration abilities of CRC cells caused by CCDC113 overexpression in vitro and in vivo. **A**, **B** qRT-PCR (**A**) and Western blotting (**B**) detected CCDC113 overexpression in SW480 cells. **C** CCK-8 assays detected cell viability of CCDC113 overexpression SW480 cells with or without 10 μM galunisertib (Gal) treatment. **D** Colony formation assays detected proliferation abilities of CCDC113 overexpression SW480 cells with or without 10 μM galunisertib treatment. Representative images were shown in left panel and statistical results were shown in right panel. **E** Transwell migration assays detected migration abilities of CCDC113 overexpression SW480 cells with or without 10 μM galunisertib treatment. Scale bars, 100 μm. Representative images were shown in left panel and statistical results were shown in right panel. **F** Wound-healing assays detected migration abilities of CCDC113 overexpression SW480 cells with or without 10 μM galunisertib treatment. Representative images were shown in upper panel and statistical results were shown in lower panel. **G**–**I** CCDC113 overexpression HCT116 cells and control cells were subcutaneously injected into BALB/c nude mice, followed by oral gavage with 75 mg/kg galunisertib or vehicle. Representative images of subcutaneous xenograft tumors were shown in (**H**). Statistical results of tumor volumes (**G**) and tumor weights (**I**) were shown (*n* = 5 per group). Data are presented as means ± SD. ***p < 0.001, **p < 0.01, *p < 0.05, ns no significance.

To investigate molecular mechanisms of CCDC113 in CRC tumorigenesis and metastasis, we performed high-throughput RNA sequencing (RNA-seq) of CCDC113 knockdown (shCCDC113) HCT116 cells and control (shNC) HCT116 cells. Volcano plot revealed 565 DEGs between shCCDC113 group and shNC group, including 148 upregulated genes and 417 downregulated genes (Fig. 7A). GO pathway enrichment analysis for DEGs in (A) revealed that DEGs were predominantly enriched in SMAD protein signal transduction, pathway-restricted SMAD protein phosphorylation, SMAD binding and transforming growth factor beta receptor activity (Fig. 7B). SMADs are key proteins in TGF-β signaling pathway [21]. KEGG enrichment showed that ECM regulators were suppressed and temporal TGF-β1 signature down was activated (Fig. 7C). TGF-β can induce EMT and excessive deposition of Extracellular Matrix (ECM), thus promoting tumor invasion and metastasis [22, 23]. Meanwhile, Gene set enrichment analysis (GSEA) showed TGF-β signaling pathway was inhibited after CCDC113 knockdown (Fig. 7D). And this result was consistent with the KEGG enrichment analysis. Correlation analysis showed CCDC113 was significantly positively correlated with TGF-β signaling pathway from TCGA database (Fig. 7E). These results suggest that CCDC113 may activate TGF-β signaling pathway to promote CRC tumorigenesis and metastasis. RNA-seq showed that expression of CD24, FERMT1, EPCAM, and SLC3A2 were decreased after CCDC113 knockdown (Fig. 7F), which were identified as TGF-β signaling pathway-related genes. CD24 was reported to regulate TGF-β1-mediated migration and EMT by regulating Src/FAK activity [24]. FERMT1 regulates TGF-β-induced EMT in breast cancer cell lines to promote breast cancer development and lung metastasis [25]. EPCAM cooperates with TGF-β signaling pathway to enhance tumorigenesis and metastasis in HCC [26]. SLC3A2 coordinates with TGF-β signaling pathway during aging [27]. Of note, correlation analysis showed positive correlations of CD24, FERMT1, EPCAM and SLC3A2 with CCDC113 in CRC (Fig. 7G). We also validated the positive correlation of CD24, FERMT1, EPCAM and SLC3A2 with CCDC113 by qRT-PCR (Fig. 7H). Importantly, CD24, FERMT1, EPCAM and SLC3A2 was also positively correlated with TGF-β signaling pathway in CRC (Fig. 7I). Moreover, according to TCGA and GTEx database, CD24, FERMT1, EPCAM and SLC3A2 are highly expressed in CRC (Fig. 7J). To further validate whether CCDC113 promotes CRC tumorigenesis and metastasis via TGF-β signaling pathway, we constructed CCDC113 overexpression SW480 cells (Fig. 8A, B). CCK-8 assays and colony formation assays indicated that CCDC113 overexpression significantly promoted proliferation abilities of SW480 cells (Fig. 8C, D). And TGF-β signaling pathway inhibitor galunisertib (Gal) could reverse these effects (Fig. 8C, D). Transwell migration assays and wound-healing assays showed galunisertib could reverse enhanced migration abilities caused by CCDC113 overexpression (Fig. 8E, F). Moreover, galunisertib could impede increased tumor growth caused by CCDC113 overexpression in vivo (Fig. 8G–I). Conclusively, CCDC113 promotes CRC tumorigenesis and metastasis via TGF-β signaling pathway.

## Discussion

CRC is one of the most common malignant cancers worldwide accounting for 10% of all cancer cases and deaths [28]. According to global cancer statistics, more than 1.9 million new cases and 935,000 deaths occurred in 2020 [29]. We previously defined several key regulators in CRC that played important roles in the regulation of CRC progress and metastasis [30, 31]. In the present study, we screened out a key regulator named CCDC113 was highly expressed in CRC. And the expression of CCDC113 had a good correlation with CRC patients’ survival. Loss of function or gain of function assays indicated that CCDC113 was crucial for CRC growth and metastasis. Thus, targeting CCDC113 may be a novel strategy for CRC intervention.

CCDC family members are widely expressed, and they play important roles in multiple biological processes, including tumor growth, invasion, metastasis and chemical sensitivity [10, 14]. They may also be potential targets for cancer therapy [10]. CCDC family members exert different functions in tumors. CCDC8 and CCDC65 have been reported to act as tumor suppressors in tumors such as gastric and lung cancer [13, 32–34]. However, other CCDC family members have been reported to promote tumorigenesis and metastasis [12, 35–37]. In the present study, we found that CCDC family member CCDC113 acted as oncogene to promote CRC tumorigenesis and metastasis. Thus, more exploration will be meaningful to define what roles CCDC family members play in tumorigenesis and metastasis.

CCDC113 was originally found in CD34^+^ hematopoietic stem/progenitor cells (HSPCs) and contains two sequences: amidation site and leucine zipper motif (LZ) [38]. CCDC113, satellite proteins HAP1 and PCM1 form centriolar satellite protein complex, and CCDC113 plays a role in primary cilia formation [15]. Ccdc113/Ccdc96 complex has been reported to regulate ciliary activity and ciliary beating [16]. CCDC113 is also identified to be key gene involved in the effect of cognitive training immediately after rTMS (rTMS-COG_0h_) on PSCI [17]. The expression of CCDC113 is higher in asthmatic nasal brushes than healthy controls [18], and CCDC113 has been reported to be predictors of omalizumab response in moderate-to-severe asthma patients [19]. It has been reported that CCDC113 can be used as diagnosis detection biomarkers of early lung cancer [20]. In the present study, we showed that CCDC113 was upregulated in CRC. CCDC113 played an important role in CRC tumorigenesis and metastasis as an oncogene. CCDC113 may be a potential biomarker and therapeutic target for CRC intervention.

Volcano plot of RNA-seq shows 565 DEGs between shNC group and shCCDC113 group (Fig. 7A). Among 148 upregulated genes, IGFBP3 (insulin-like growth factor-binding protein 3), a tumor suppressor, plays an important role in CRC tumorigenesis [39]. PLK2 (polo-like kinase 2) plays important roles in CRC progression and invasive [40]. Among 417 downregulated genes, high expression of non-mitochondrial C1QBP is correlated with high grade CRC [41]. And expression of vimentin (VIM) is correlated with CRC patients’ poor prognosis [42]. Moreover, phosphorylation of VIM promotes growth of CRC metastasis tumors [43]. These suggest that CCDC113 knockdown inhibit proliferation and metastasis abilities of CRC cells. These are consistent with our results that CCDC113 is an oncogene.

TGF-β signaling pathway plays important roles in early embryonic development, tissue and organ formation, immune supervision, tissue repair and tumorigenesis [44]. In pathological conditions, overexpression of TGF-β leads to EMT, ECM deposition and cancer-associated fibroblasts (CAF) formation, leading to fibrosis disease and cancer [45]. EMT is a phenotypic plastic process that confers epithelial cell migration and invasion properties during development, fibrosis and cancer [46–48]. We showed that expression of MMP2 increased after CCDC113 overexpression. Also, CCDC113 expression was positively correlated with EMT according to our correlation analysis. ECM genes upregulated in cancer is correlated with the activation of TGF-β signaling in cancer-associated fibroblasts [49]. We found ECM regulators were suppressed after CCDC113 knockdown according to KEGG analysis. The downstream effectors of classical TGF-β signaling pathway are SMAD transcription factors [21, 50]. We found DEGs between shCCDC113 group and shNC group were predominantly enriched in SMAD signaling. We also examined SMAD2 and SMAD3 expression in CCDC113 knockdown HCT116 cells. We found the expression of SMAD2 and SMAD3 decreased in CCDC113-depleted HCT116 cells (Fig. S1C). Also, GSEA and correlation analysis showed positive correlations between CCDC113 and TGF-β signaling pathway. We showed CD24, FERMT1, EPCAM and SLC3A2 were positively correlated with CCDC113 according to RNA-seq analysis, correlation analysis and qRT-PCR. Also, CD24, FERMT1, EPCAM and SLC3A2 were positively correlated with TGF-β signaling pathway. Blocking TGF-β signaling pathway could inhibit increased proliferation and migration abilities of CCDC113 overexpression CRC cells in vitro and in vivo. Thus, CCDC113 may promote SMAD2 and SMAD3 expression, thereby activating TGF-β signaling pathway and promoting CRC tumorigenesis and metastasis.

In conclusion, exploring how key genes regulate CRC tumorigenesis and metastasis has tremendous biological and clinical importance. To elucidate it, we performed bioinformatics analysis to screen key genes in GEO CRC databases. We demonstrated that CCDC113 promoted CRC tumorigenesis and metastasis via TGF-β signaling pathway.

However, our study has some drawbacks. More experimental validation is necessary to elucidate how CCDC113 promoting CRC tumorigenesis and metastasis via TGF-β signaling pathway.

## Methods

### Gene ontology and pathway enrichment analysis

GO enrichment analysis of DEGs and PPI sub-network genes were performed using the enrichGO function in clusterProfiler package (https://github.com/YuLab-SMU/clusterProfiler). The functional categories considered encompassed biological processes (BP), cellular components (CC) and molecular functions (MF). The organism reference was specified as “org.Hs.eg.db”. Terms meeting the criteria of *p* < 0.05 and *p* Adjust Method = “BH” were selected. Similarly, KEGG pathway analysis were conducted utilizing clusterProfiler (enrichKEGG). EnrichGO and enrichKEGG functions were used to implement enrichment analysis. The *p* values were calculatesd using the hypergeometric distribution.

### Kaplan-Meier survival analysis

GDC TCGA colon cancer cohort file and clinical survival data file were downloaded from the UCSC Xena database (http://xena.ucsc.edu/). Tumor patients were separated into two groups, the optimal cutoff value was determined using the surv_cutpoint and surv_categorize function of the R package survminer (v0.4.9). Kaplan-Meier curves for overall survival were calculated using the Surv() function, log-rank tests comparing survival curves calculated using the survdiff() function and survival curves were computed using the “survfit” function. And then we used ggsurvplot from the survminer R package to generate the survival plot.

### Protein and protein interaction (PPI) network

PPI network of 222 common DEGs was constructed using Search Tool for the Retrieval of Interacting Genes (STRING, https://string-db.org). PPI network was generated based on an interaction combined score > 0.4. Subsequently, interaction information network was visualized using Cytoscape software (v3.9.1). Molecular Complex Detection (MCODE) plugin was applied to select the sub-networks from PPI network (MCODE Score=7.269).

### Cell culture

NCM460 cells (human normal colonic epithelial cell), HEK293T cells and human CRC cell lines (HCT116, RKO, LoVo, HT29, SW620 and SW480) were maintained in cell incubator (Thermo Fisher Scientific) with 37°C and 5% CO_2_. Cells were routinely cultured in DMEM medium (VivaCell, #C3113-0500) or 1640 medium (VivaCell, #C3010-0500) supplemented with 10% FBS (fetal bovine serum) (Lonsera, #S711-001S) and 1% Penicillin-Streptomycin antibiotics solution (Beyotime, #C0222). All cell lines were tested for mycoplasma contamination and authenticated using an STR profiling.

### Knockdown and overexpression plasmid construction

We looked for gene targets on Invitrogen Block-iT RNAi Designer (https://rnaidesigner.thermofisher.com/rnaiexpress) and designed shRNAs with a fixed structure (target sequences: shCCDC113-1: 5′-GCGACCTCCACGATTATCA-3′; shCCDC113-2: 5′-GATAGCAGAGATGTCCTTA-3′). shRNA primers were synthesized by BGI Tech Solutions (Beijing Liuhe). shRNAs were linked to enzyme-digestion pSicoR PGK puro plasmid (Addgene, #12084). To construct CCDC113 overexpression plasmid, we cloned CCDC113 gene sequence to pLVX-IRES-Zsgreen1 plasmid. The recombined plasmids were extracted using EndoFree Plasmid Midi Kit (Cwbio, #CW2105).

### Lentiviral infection

CCDC113 knockdown or CCDC113 overexpression plasmid and control plasmid were transfected into HEK293T cells with lentiviral packaging plasmids psPAX2 (Addgene, #12260) and pMD2.G (Addgene, #12259) using jetPRIME reagent (Polyplus, #101000046) according to protocol of manufacturer. HEK293T cells were seeded in 6-well plates (NEST, #703001) before transfection. The transfection mix was discarded after 6 h and replaced with fresh DMEM medium containing 10% FBS. Viral fluids were harvested at 48 h and filtered by 0.45 μm syringe filter (BioFil, #FPV403030). HCT116 and RKO cells were seeded in 6-well plates the day before infection and cultured in DMEM or 1640 medium with 10% FBS until cells reached 40% confluency. The original medium was replaced with the filtered virus fluids and polybrene (Yeasen, #40804ES86). Virus fluids were discarded after 6 h and replaced with fresh DMEM or 1640 medium containing 10% FBS. After 48 h, cells were selected by puromycin (1 mg/ml for HCT116 cells and 1.5 mg/ml for RKO cells) for 3-4 days to generate stable CCDC113 knockdown cells. And successful construction of CCDC113 overexpression cells were confirmed by observing fluorescence of HCT116, RKO and SW480 cells under fluorescence microscope (Nikon Eclipse Ti2, Japan). Efficiency of CCDC113 knockdown or overexpression was detected by qRT-PCR and Western blotting.

### Cell counting kit-8 (CCK-8) assay

Proliferation abilities of CRC cells were detected by cell counting kit-8 (APExBIO, #K1018). Cells (2×10^3 cells per well per 100 μL medium) of logarithmic growth phase were seeded into 96-well plates. Then 10 μL CCK-8 solution was added into each well at 0, 24, 48, 72, 96 hours, followed by incubation under dark place at 37°C for 1 h. Absorbance value at wavelength of 450 nm (OD450) was detected at corresponding time by microplate reader (Spectramax Absorbance Reader CMax Plus, Molecular Devices, USA).

### Colony formation assay

CRC cells (5 × 10^2^ cells per well) were seeded into 6-well plates and incubated at 37 °C for 10-18 days. Cells were fixed with 4% paraformaldehyde for 30 minutes and stained with 0.1% crystal violet for 20 minutes. Colony numbers were counted by Image J and recorded.

### Transwell migration assay

CRC cells (2 × 10^4^ cells) were resuspended in 200 μL medium without FBS and seeded into 8 μm pore upper chambers of transwell chambers (Corning, #3422), while lower chambers were filled with medium containing 20% FBS. After 48 h, CRC cells were fixed with 4% paraformaldehyde for 30 minutes and stained with 0.1% crystal violet for 20 minutes. Images were taken under microscope Eclipse Ti2 (Nikon, Japan). Migration cells were counted by Image J and recorded.

### Wound-healing assay

CRC cells were seeded in 6-well plates and grew until confluency. Confluent cells were scraped by 10 μL pipette tip (Axygen, #AXYT300). After 24 h, 36 h and 48 h, cells migrated to the wound and the scratched area was taken by inverted microscope Eclipse Ti2 (Nikon, Japan). The migration distance was measured by Image J and migration rate was obtained by formula: (Width 0 h − Width 24/36/48 h) / Width 0 h.

### Apoptosis assay

Apoptotic rate of CRC cells was detected by apoptosis detection kit (Vazyme, #A214) and (Yeasen, #40302ES60). After treatment with EDTA-free trypsin, cells were collected by centrifugation at 4 °C for 5 min at 300 g. After washing cells twice with pre-cooled PBS, 100 μL 1×Binding Buffer was added to 1-5×10^5^ cells. Then cells were incubation with 5 μL Annexin V-FITC/APC and 10 μL PI Staining Solution at room temperature for 10-15 min in the dark. Finally, 400 μL 1×Binding Buffer was added and mixed well for detection by flow cytometry within 1 hour.

### RNA isolation and quantitative real-time PCR (qRT-PCR)

Total RNA from whole cell lysates or tissues were isolated by Total RNA Extraction Kit (Beibei biotechnology, #082001) and TRIzol reagent (Sigama, #93289) according to the protocol of manufacturer. 1 μg of total RNA was reverse transcribed to cDNA by HiScript III RT SuperMix for qRT-PCR (+gDNA wiper) (Vazyme, #R323). qRT-PCR was performed in CFX96TM Real-Time System (Bio-RAD, #CT032238). Each reaction was assayed in 20 μL reaction system containing AceQ Universal SYBR qRT-PCR Master Mix System (Vazyme, #Q511), primers, ddH_2_O and template DNAs. The Ct values obtained from samples were compared using 2^−ΔΔCt^ method. β-actin served as internal reference genes. The following primers were used: CCDC113-Forward: 5’-TCCGACTCCCATGAAGGGT-3’; CCDC113-Reverse: 5’-CTGATAATCGTGGAGGTCGCT-3’; β-actin-Forward: 5’-GGCGGCACCACCATGTACCC-3’; β-actin-Reverse: 5’-AGGGGCCGGACTCGTCATACT-3’; E-cadherin-Forward:5’-CAGCACGTACACAGCCCTAA-3’; E-cadherin-Reverse: 5’-TGGGAGGAAGGTCTGCATCA-3’; N-cadherin-Forward:5’-ACAGTGCAGTCTTATCGAAGGAT-3’; N-cadherin-Reverse: 5’- GCTTCTCACGGCATACACCA-3’; VIMENTIN-Forward:5’-GCTAACCAACGACAAAGCCC-3’; VIMENTIN-Reverse:5’-CGTTCAAGGTCAAGACGTGC-3’;TWIST1-Forward:5’-CTCGGACAAGCTGAGCAAGA-3’; TWIST1-Reverse: 5’-CTCCATCCTCCAGACCGAGA-3’; MMP2-Forward:5’-CTTCCAAGTCTGGAGCGATGT-3’; MMP2-Reverse: 5’-TACCGTCAAAGGGGTATCCAT-3’; MMP9-Forward:5’-TACCACCTCGAACTTTGACA-3’; MMP9-Reverse: 5’-AGGGCGAGGACCATAGAG-3’; CD24-Forward:5’-CCAGTGAAACAACAACTGGAACT-3’; CD24-Reverse: 5’-GCAGAAGAGAGAGTGAGACCAC-3’; FERMT1-Forward:5’-ACAGCCCTTTGACGGAACAA-3’; FERMT1-Reverse:5’- TCCATAAGGGAGCGTGAGGA-3’; EPCAM-Forward:5’-CCGCAGCTCAGGAAGAATGT-3’; EPCAM-Reverse:5’-GCTCTCATCGCAGTCAGGAT-3’; SLC3A2-Forward:5’- GGGCGTCTCGATTACCTGAG-3’; SLC3A2-Reverse: 5’-GTTCTCACCCCGGTAGTTGG-3’; SMAD2-Forward:5’-AGAGATATGGCTGGCACCCT-3’; SMAD2-Reverse:5’-TGCCTTCGGTATTCTGCTCC-3’; SMAD3-Forward:5’-CATCCTGCCTTTCACTCCCC-3’; SMAD3-Reverse:5’-CTGGGGATGGTGATGCACTT-3’.

### Western blotting

Cells and tissues were lysed by RIPA buffer (Beyotime, #P0013B) with protease inhibitor PMSF (Solarbio, #P0100). Proteins were harvested and separated by SDS-polyacrylamide gel electrophoresis (PAGE) and electrotransferred onto polyvinylidene fluoride (PVDF) membrane (Merck millipore, #IPVH00010). After incubating with primary and secondary antibodies, protein expression was visualized using chemiluminutesescent reaction kit of BeyoECL Star (Beyotime, #P0018AS) by enhanced chemiluminutesescent system BG-gds AUTO 720 (Baygene Biotech, China). GAPDH served as internal reference protein. The following primary antibodies were used: anti-CCDC113 (1:1000, GeneTex, #GTX12-0455), anti-BAX (1:1000, Proteintech, #50599-2-Ig), anti-MMP2 (1:1000, Proteintech, #10373-2-AP), anti-BCL2 (1:1000, Proteintech, #12789-1-AP) and anti-GAPDH (1:1000, Santa Cruz, #sc-47724). The following secondary antibodies were used: goat anti-rabbit secondary antibody (1:3000, ZSGB-Bio, #ZB-2301) and goat anti-mouse secondary antibody (1:3000, ZSGB-Bio, #ZB-2305).

### Immunofluorescence

CRC cells were seeded on slides (Biosharp, #BS-14-RC) for overnight and fixed with 4% paraformaldehyde at 4 °C for 20 minutes. Between each steps described below, cells were rinsed 2 minutes for three times by PBST buffer. Cells were blocked with 10% Donkey Serum (Solarbio, #SL050) for 1 h at room temperature. Then, cells were incubated overnight with CCDC113 antibody (1:200, GeneTex, #GTX120455) in a humidified chamber at 4 °C. Next, cells were incubated with secondary antibody IFKine™ Red Donkey Anti-Rabbit IgG (1:200, Abbkine, #A24421) kept in dark place at room temperature for 1 h. Then slides were stained with DAPI (1:1000, Sigma, #D9542-1MG) for 5 minutes at room temperature and sealed with antifading mounting medium (Solarbio, #S2100). Fluorescence confocal microscopy (ZEISS, LSM880, Germany) was used for imaging.

### Animal experiments

Four-week-old female BALB/c nude mice were purchased from SPF Biotechnology Co.,Ltd (Beijing, China). Mice were divided into appropriate groups based on the experimental design. For subcutaneous xenograft tumor model, HCT116 cells (2 × 10^6^ cells per mouse) were resuspended in 100 μL sterile PBS and injected into the upper back of BALB/c nude mice. Then tumor volumes were measured every 3 days. Tumor volumes were calculated using the following formula: volume (mm^3^) = length × width^2^/2. Part of tumor tissues were used for protein extraction to detect CCDC113 expression; Part of tumor tissues were fixed with 4% paraformaldehyde for 24 h and embedded with paraffin wax for immunohistochemistry. For tail vein metastasis model, the BALB/c nude mouse tails were wiped with alcohol and then HCT116 cells were injected into the tail vein through an insulin syringe. In knockdown group, CCDC113 knockdown or control HCT116 cells (5 × 10^6^ cells per mouse) suspended in 100 μL sterile PBS (*n* = 3 per group) were injected for 42 days. In overexpression group, CCDC113 overexpression or control HCT116 cells (2 × 10^6^ cells per mouse) suspended in 100 μL sterile PBS were injected (*n* = 4 per group) for 28 days. Their livers were surgically excised and photographed when reach endpoint. Subsequently, liver tissues were fixed with 4% paraformaldehyde for 24 h and embedded with paraffin wax. Hematoxylin and eosin (HE) staining was performed to analyze metastasis ability by counting liver metastatic nodule numbers and liver metastatic nodule area. IHC was also performed to analyze CCDC113 and Ki67 expression in liver metastatic nodules. For galunisertib treatment in vivo experiments, HCT116 cells (3 × 10^6^ cells per mouse) were resuspended in 100 μL sterile PBS and injected into the upper back of BALB/c nude mice. Mice were treated with vehicle or 75 mg/kg galunisertib by oral gavage. The tumor volumes were measured every 3 days. Tumor volumes were calculated using the following formula: volume (mm^3^) = length × width^2^/2. No statistical methods were applied to choose the number of mice; no randomization method was used to choose the animals for this analysis; no blind analysis was applied.

### Immunohistochemistry (IHC)

Tissues were fixed with 4% paraformaldehyde for 24 h and embedded with paraffin wax. Then they were cut into 5 μm thick slices. Then, slices were dewaxed in xylene and rehydrated in 100%, 95%, 80% of alcohols. Tissues were incubated with 3% hydrogen peroxide solution for 10 minutes to block endogenous peroxidase activity. Between each steps described below, slices were rinsed 2 minutes for three times by PBS buffer. Then they were blocked by 10% Donkey Serum (Solarbio, #SL050) at room temperature for 30 minutes. Antigen was retrieved by heating slices at 100°C for 15 minutes in EDTA solution. Next, slices were incubated with CCDC113 antibody (1:300, GeneTex, #GTX120455) or Ki67 antibody (1:2000, Proteintech, #27309-1-AP) at 4°C overnight. After washing with PBS, slices were incubated with goat anti-rabbit secondary antibodies (1:3000, ZSGB-Bio, #ZB-2301) at room temperature for 1 h. Then, 3,3’-Diaminutesobenzidine (DAB) Horseradish Peroxidase Color Development Kit (ZSGB-BIO, #ZLI-9019) was used as chromogen and hematoxylin was applied as nuclear counterstain. Finally, the slices were sealed by neutral balsam after dehydration and clearing. The slides were observed using SLIDEVIEW VS200 slide scanner (Olympus, Japan).

### Statistical analysis

Each experiment was conducted at least three times independently and results were analyzed using SPSS 26.0, R 4.3.2 and GraphPad Prism. The numerical data were with normal distribution and the variance was similar between the groups that were being statistically compared. *P* values were calculated by two-tailed Student’s t-test for comparing two groups or one way ANOVA for comparing three groups. Data that didn’t follow normal distribution were analyzed by nonparametric test. The variance wasn’t similar between groups were analyzed by Welch’s correction. No statistical methods were applied to predetermine the sample size. Blinding was not used for data collection. Gene correlation analysis in R was conducted utilizing Pearson’s method. Single-sample GSEA enrichment scores were calculated for Hallmark_TGF_BETA_SIGNALING using R package GSVA. *P* < 0.05 was considered statistically significant (*p < 0.05, **p < 0.01, ***p < 0.001, ns means no statistic difference).

## Supplementary information

Supplementary Figure 1

## Data Availability

All data are available upon reasonable request.
