# Supplementary material for: CCDC113 promotes colorectal cancer tumorigenesis and metastasis via TGF-β signaling pathway
Source: Cell Death Dis. 2024 Sep 11;15(9):666. doi: 10.1038/s41419-024-07036-3 (PMC11390942; doi:10.1038/s41419-024-07036-3)
Supplement: Supplementary file 1 — Supplementary Figure 1 [file 41419_2024_7036_MOESM1_ESM.pdf]

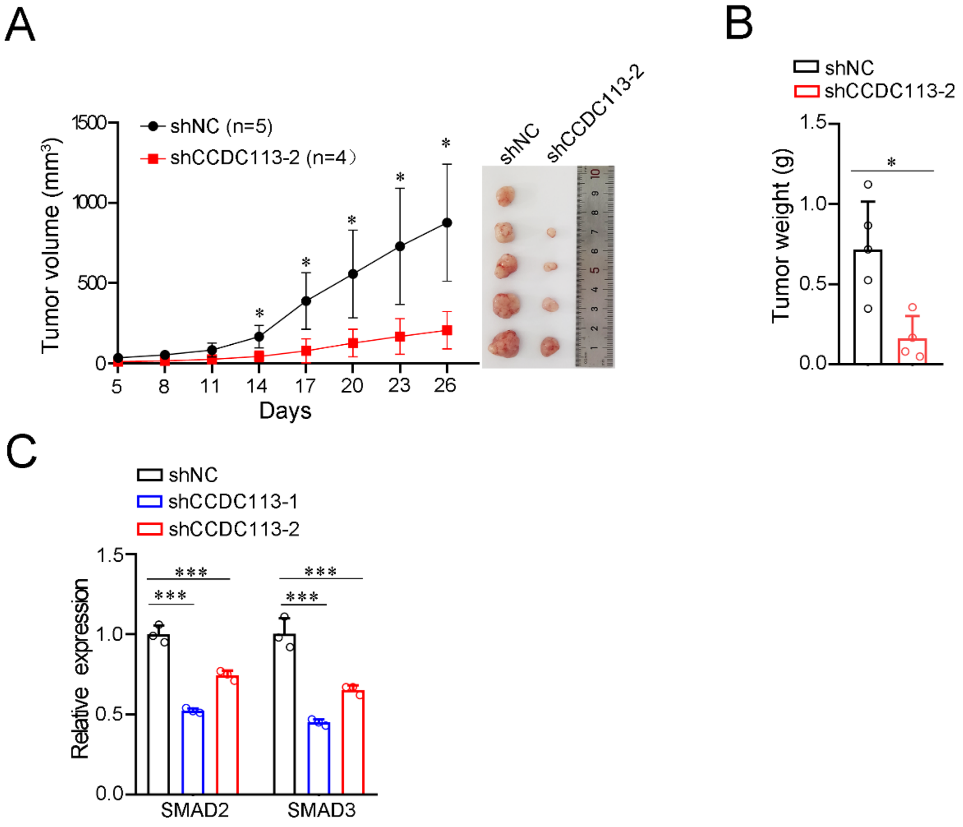

Supplementary Figure legends

Figure S1. *In vivo* and *in vitro* assays of CCDC113 knockdown HCT116 cells. (A-B) CCDC113 knockdown HCT116 cells and control cells were subcutaneously injected into BALB/c nude mice for 26 days. Tumor volumes (A), images of subcutaneous xenograft tumors (A) and tumor weights (B) were shown. (C) *SMAD2* and *SMAD3* mRNA levels in shCCDC113 and shNC HCT116 cells were detected by qRT-PCR. Data are presented as means ± SD. ***$p < 0.001$, *$p < 0.05$.
